# Dose-Dependent Effects of Dietary Fat on Development of Obesity in Relation to Intestinal Differential Gene Expression in C57BL/6J Mice

**DOI:** 10.1371/journal.pone.0019145

**Published:** 2011-04-25

**Authors:** Nicole J. W. de Wit, Mark V. Boekschoten, Eva-Maria Bachmair, Guido J. E. J. Hooiveld, Philip J. de Groot, Isabel Rubio-Aliaga, Hannelore Daniel, Michael Müller

**Affiliations:** 1 Netherlands Nutrigenomics Centre, Top Institute Food and Nutrition, Wageningen, The Netherlands; 2 Nutrition, Metabolism and Genomics Group, Division of Human Nutrition, Wageningen University, Wageningen, The Netherlands; 3 Rowett Institute of Nutrition and Health, University of Aberdeen, Aberdeen, United Kingdom; 4 Molecular Nutrition Unit, Research Center for Nutrition and Food Sciences, Technische Universität München, Freising-Weihenstephan, Germany; University of Tor Vergata, Italy

## Abstract

Excessive intake of dietary fat is known to be a contributing factor in the development of obesity. In this study, we determined the dose-dependent effects of dietary fat on the development of this metabolic condition with a focus on changes in gene expression in the small intestine. C57BL/6J mice were fed diets with either 10, 20, 30 or 45 energy% (E%) derived from fat for four weeks (n = 10 mice/diet). We found a significant higher weight gain in mice fed the 30E% and 45E% fat diet compared to mice on the control diet. These data indicate that the main shift towards an obese phenotype lies between a 20E% and 30E% dietary fat intake. Analysis of differential gene expression in the small intestine showed a fat-dose dependent gradient in differentially expressed genes, with the highest numbers in mice fed the 45E% fat diet. The main shift in fat-induced differential gene expression was found between the 30E% and 45E% fat diet. Furthermore, approximately 70% of the differentially expressed genes were changed in a fat-dose dependent manner. Many of these genes were involved in lipid metabolism-related processes and were already differentially expressed on a 30E% fat diet. Taken together, we conclude that up to 20E% of dietary fat, the small intestine has an effective ‘buffer capacity’ for fat handling. From 30E% of dietary fat, a switch towards an obese phenotype is triggered. We further speculate that especially fat-dose dependently changed lipid metabolism-related genes are involved in development of obesity.

## Introduction

Excessive intake of dietary fat is known to be a substantial contributing factor to the development of obesity in Western society. Many mouse studies have been performed to study the effect of dietary fat on the etiology of obesity, a major risk factor of the metabolic syndrome [1], [2], [3], [4]. For this, most studies compared the effects of two diets with a low versus high fat-content. Moreover, these differences in fat-dose are mostly quite extreme, such as 5–10 energy percent (E%) fat versus 30–45E% fat or even higher. Dose-dependent effects of dietary fat on the development of obesity are rarely investigated.

Whereas organs, such as liver, muscle and white adipose tissue are frequently studied in relation to dietary fat-induced obesity and insulin resistance, the intestine is mostly neglected [5], [6], [7], [8]. However, there is growing evidence that the small intestine as an endocrine organ also contributes to the etiology of the metabolic syndrome. Several studies have previously shown that dietary fat-induced changes in intestinal gene expression might be related to obesity [9], [10]. In these studies, the effects of low fat (5–10E%) and high fat (30–45E%) diets were compared, but dose-dependent effects of dietary fat have never been studied. Therefore in this study, we used diets with 10E% (control diet), 20E%, 30E% and 45E% of dietary fat to determine dose-dependent fat-effects on development of obesity in C57Bl/6J mice and determined differential gene expression in the proximal, middle and distal part of the small intestine of these mice.

## Materials and Methods

### Ethics statement

The institutional and national guidelines for the care and use of animals were followed and the experiment was approved by the Local Committee for Care and Use of Laboratory Animals at Wageningen University.

### Animals and diets

The study described here is part of the Nugo Proof of Principle study package as described by Baccini et al. [11].

Male C57BL/6J mice were obtained from Charles River (Maastricht, The Netherlands) at three weeks of age. The mice were housed in pairs. At twelve weeks of age, all mice received control diet as a run-in for four weeks. After the run-in period mice were divided in four groups that received 45, 30, 20, or a control diet of 10 energy % (10E%) of dietary fat (n = 10 per group). Palm oil was the main fat source in the diets. The only other variable in the diets was the amount of corn starch (Table S1). Body weight and food intake were measured weekly. After four weeks of diet intervention, mice were fasted for five hours and subsequently anaesthetized with a mixture of isoflurane (1.5%), nitrous oxide (70%), and oxygen (30%). Mice were killed by cervical dislocation. For RNA isolation small intestine was excised, divided in three equal parts, cut open longitudinally, and washed with PBS. The mucosa was scraped and snap frozen in liquid nitrogen.

### Transcriptomics analysis

Total RNA was extracted from intestinal scrapings with TRIzol reagent (Invitrogen, Carlsbad, CA), treated with DNase and purified on columns using the RNeasy Mini Kit (Qiagen, Venlo, The Netherlands). RNA integrity was checked on an Agilent 2100 Bioanalyzer (Agilent Technologies, Amsterdam, The Netherlands) with 6000 Nano Chips. RNA was judged as suitable only if samples showed intact bands of 18S and 28S ribosomal RNA subunits, displayed no chromosomal peaks or RNA degradation products, and had a RNA integrity number (RIN) above 8.0.

For each individual mouse (n = 10 per diet group), total RNA (5 µg) of the proximal, middle and distal part of the small intestine was labeled using the Affymetrix One-Cycle Target Labelling Assay kit (Affymetrix, Santa Clara, CA). Each individual labeled RNA sample (n = 120), was hybridized on a Affymetrix NuGO mouse array, washed, stained, and scanned on an Affymetrix GeneChip 3000 7G scanner. The NuGO arrays are custom designed Affymetrix GeneChip arrays, designed by the European Nutrigenomics Organisation (NuGO) and manufactured by Affymetrix. These arrays contain in part common probe sets that are also present on standard Affymetrix arrays and in part newly designed probe sets (Gene Expression Omnibus platform GPL7440).

Packages from the Bioconductor project, integrated in an in-house developed online management and analysis database for multiplatform microarray experiments, were used to analyze scanned arrays [12]. Probesets were redefined according to Dai et al. using remapped CDF version 11.0.2 based on the Entrez Gene database [13]. The Nugo Mouse GeneChip arrays target 15,240 unique gene identifiers. GC-robust multi-array (GCRMA) analysis was used to obtain expression values [14]. Only genes that had an unlogged intensity above five on at least five arrays were considered for analysis. The normalization and filtering procedure was performed separately for each of the three parts of the small intestine. In each part of the intestine, approximately 11,000 genes were detected. The Bioconductor R package Linear models for microarray data (LIMMA) was used to identify differentially expressed genes, based on the 10 replicate microarrays per diet group, per part of the small intestine. [15]. Obtained p-values were corrected for multiple testing using a false discovery rate (FDR) method [16]. Genes that met the cut-off of FDR q-value<0.05 were considered to be significantly regulated. All microarray data are MIAME compliant and array data have been submitted to the Gene Expression Omnibus (accession number GSE26300).

### Statistical analysis

Physiological data are reported as the mean ± the standard error (SE). The differences between the mean values were tested for statistical significance by a one-way ANOVA with an additional Bonferroni post-hoc test (PASW Statistics 17.0 software, SPSS Inc., Chicago, Illinois, USA). P-values<0.05 are considered statistically significant.

## Results

### Food intake and weight gain

**Figure 1:**
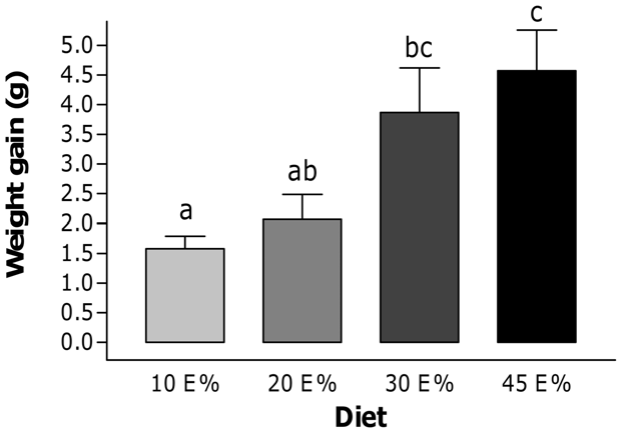
Body weight gain and blood glucose levels. Body weight gain of C57BL/6J mice fed a 10E%, 20E%, 30E% or 45E% fat diet for four weeks. The data are given as means ± SE. a, b, c: bars with superscripts without a common letter differ, p<0.05.

To determine dose-dependent effects of dietary fat on development of obesity, C57Bl/6J mice were fed four diets containing different amounts of dietary fat, namely 10E%, 20E%, 30E% and 45E% of palm oil, for four weeks. Food intake was not significantly different between groups, although there was a slightly increased energy intake as the amount of fat in the diet increased. In comparison to the 10E% fat diet serving as a reference, weight gain was not increased significantly on the 20E% fat diet whereas animals on 30E% and 45E% fat diets displayed significantly higher weight gains (Figure 1). Therefore, the main shift towards an obese phenotype occurs with diets of 20E% to 30E% of fat, which is not significantly worsened with higher levels of dietary fat.

### Differential gene expression in the small intestine

**Figure 2:**
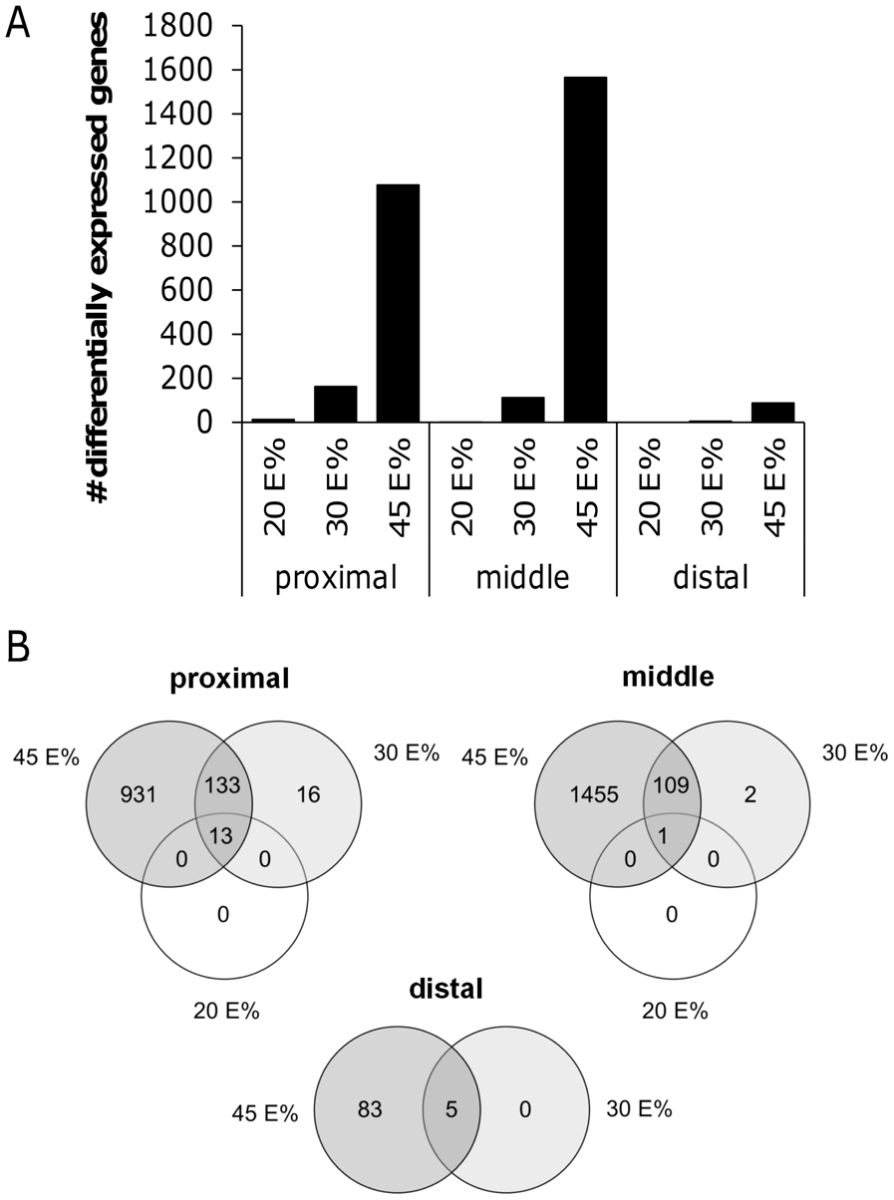
Differential gene expression along the longitudinal axis of the small intestine. For the proximal, middle and distal part of the small intestine, the numbers of genes that are differentially expressed on a 20E%, 30E% and 45E% fat diet compared to the control 10E% fat diet are plotted (A). Venn diagrams display the overlap of differentially expressed genes between the 20E%, 30E% and 45E% fat diets for each part of the small intestine (B).

In a previous study we have shown that the intestine plays an important role in the development of dietary fat-induced metabolic syndrome [10]. A 45E% fat diet intervention induced clear effects on intestinal gene expression that could be linked to obesity. As we here observed dose-dependent effects of dietary fat on development of obesity, we analyzed gene expression changes in mucosal scrapings of the proximal, middle and distal part of the small intestine. Differential gene expression was determined for each part of the small intestine by comparing the gene expression profiles of mice (n = 10 per diet group) fed the 20E%, 30E% and 45E% fat diets with the reference group (10E% fat diet). We found a gradient in numbers of differentially expressed genes, with hardly any changes in tissues of animals on the 20E% fat diet and the highest number of differentially expressed genes on the 45E% fat diet (Figure 2A). Remarkably, for the 20E% and 30E% fat diet groups, changes in gene expression were mainly detected in the proximal small intestine, whereas on the 45E% fat diet the effect was most pronounced in the middle part. For all diet groups hardly any changes in gene expression could be detected in the distal small intestine. Although the numbers of differentially expressed genes were much lower in the 20E% and 30E% diet groups, we found a substantial overlap with genes that were also differentially expressed on the 45E% fat diet (Figure 2B) and this occurred in all parts of the small intestine.

**Figure 3:**
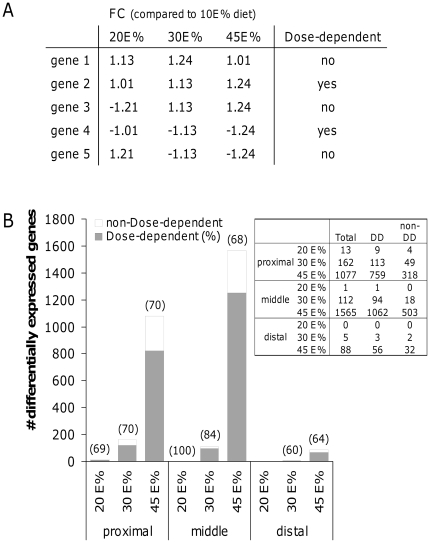
Fat-dose dependent regulation of gene expression in the small intestine. By applying specific inclusion criteria (A), fat-dose dependent regulation of gene expression was determined for the proximal, middle and distal part of the small intestine (B). In brackets, the percentages of fat-dose dependently changed genes are shown as part of total differentially expressed genes on the 20E%, 30E% and 45E% fat diets compared to the control 10E% fat diet. FC: fold changes, DD: dose-dependent, non-DD: non-dose-dependent.

**Figure 4:**
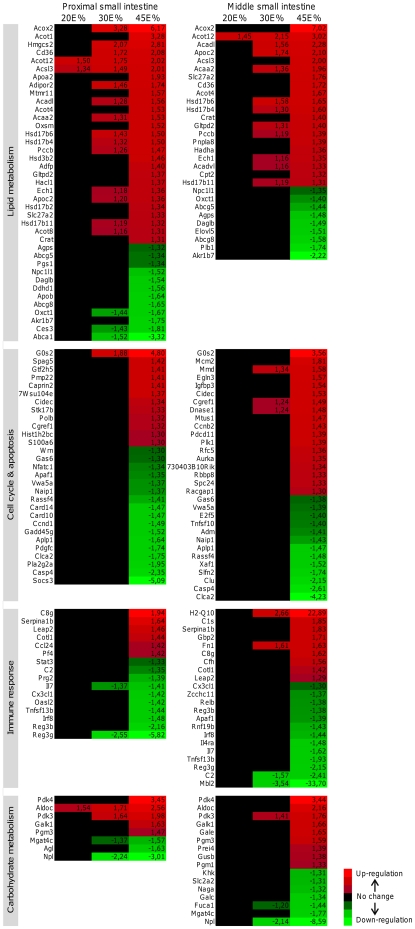
Heat map diagrams of fat-dose dependently changed genes, categorized according to their biological function. Fat-dose dependently changed genes were clustered in a heat map diagram for the proximal and middle part of the small intestine, based on their GO Biological Processes annotation. Genes involved in lipid metabolism, cell cycle/apoptosis, immune response and carbohydrate metabolism were highly overrepresented. The heat maps display fold changes of differential gene expression induced by the 20E%, 30E% and 45E% fat diets compared to the control 10E% fat diet (only genes with fold changes >1.3 or <−1.3 are shown). Red and green indicate (gradual) up- and down-regulation, respectively, whereas black means no change in gene expression.

**Figure 5:**
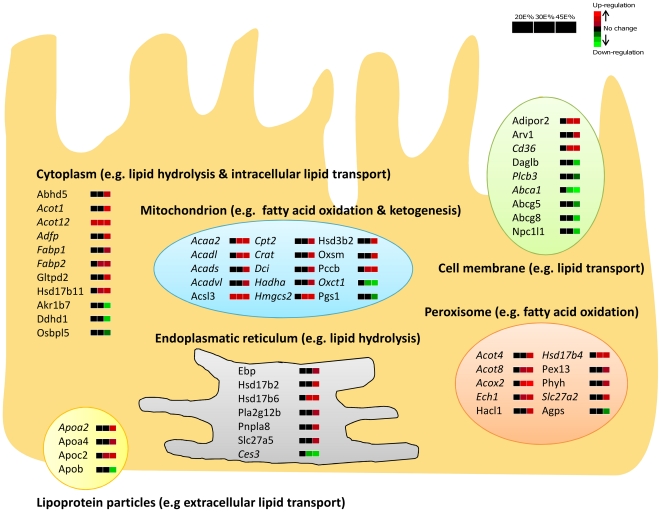
Cellular localization and specific lipid metabolism-related function of fat-dose dependently changed genes. Fat-dose dependently changed genes involved in lipid metabolism (GO Biological Processes) in the proximal part of the small intestine were categorized and visualized based on their cellular localization (GO Cellular Localization) and the specific lipid-metabolism related processes that occur in these cellular compartments. The heat maps next to the genes indicate their fat-dose dependent regulation. Red and green indicate (gradual) up- and down-regulation, respectively, whereas black means no change in gene expression. Pparα target genes are indicated in italics.

Next, we determined whether the up- or down-regulation that we found for the differentially expressed genes was dose-dependent. Therefore, we selected the genes with a significant difference in expression level (q-value<0.05) on at least one of the diets and analyzed dose-dependent changes based on the fold changes as determined by LIMMA analysis. An example is shown in figure 3A. Note that for all groups the fold changes were in the same direction (up- or down-regulation), but in the 20E% and 30E% fat diet groups not all differential expression reached significance. Remarkably, we found that in general approximately 70% of the total number of differentially expressed genes showed a dose-dependent fat effect (Figure 3B). Next, we determined in which biological processes these dose-dependently changed genes are involved based on their Gene Ontology (GO Biological Processes). This revealed that many of the genes are involved in lipid metabolism, cell cycle, immune response and carbohydrate metabolism, especially in the proximal and middle part of the small intestine (Figure 4). For lipid metabolism, a substantial number of genes already showed a significant up- or down-regulation in the 30E% fat diet group, whereas for the other biological processes significant changes were mainly restricted to the 45E% fat diet group. Differentially expressed genes involved in lipid metabolism in the proximal small intestine were further analyzed with respect to their predominant cellular localization (GO Cellular Localization) and classified by their functions within lipid metabolism (Figure 5). This analysis revealed that most cholesterol transporters showed a dose-dependent down-regulation (e.g. *Abcg5, Abcg8, Abca1 and Npc1l1*) and genes playing a role in fatty acid catabolism, especially ketogenesis and mitochondrial/peroxisomal fatty acid oxidation, were dose-dependently up-regulated (e.g *Acaa2*, *Cpt2*, *Hmgcs2*, *Acot4/8*, *Acox2*). Moreover, we found that from all dose-dependently changed genes in the proximal and middle small intestine, about 40% were peroxisome proliferator-activated receptor alpha (Pparα) target genes, according to Bunger et al. [17]. Figure 5, in which Pparα target genes are depicted in italics, shows that especially fatty acid oxidation in mitochondria and in peroxisomes are under control of Pparα that is considered to work as a fat sensor activated by fatty acids. Our data now indicate that Pparα activation in the intestine occurs in a dose-dependent manner related to dietary fat intake. For cell cycle, immune response and carbohydrate metabolism, the gene expression changes did not direct towards a clear elevation or repression of these biological processes.

## Discussion

In this study, we analyzed the dose-dependent effects of dietary fat on body weight gain, which is linked to development of diet-induced obesity. We found that a subtle elevation of dietary fat in the diet (10 E% to 20E%) had hardly any effect on development of the obese phenotype. Also on gene expression levels, we found minimal changes in the small intestine induced by the 20E% fat diet. This indicates that this subtle elevation of dietary fat has no substantial effect on the small intestine; the intestine seems to have a certain ‘buffer-capacity’ that enables the tissue to handle a limited amount of fat without changing biological processes related to metabolism, cell cycle and immune response. From 30E% of dietary fat we observed a significant increase of body weight with concomitant changes in gene expression in the small intestine. Genes with changed mRNA levels were predominantly related to lipid transport (e.g. *Fabp2*, *CD36*, *ApoC2*) and fatty acid catabolism (e.g. *Hmgcs2*, *Acox2*, *Acadl*) which all represent processes known to be regulated by Pparα [17]. This implies that on the 30E% fat diet the small intestine needs to adjust its lipid metabolism-related processes for proper handling of the overload of dietary fat that reaches the intestine and that Pparα plays an important regulatory role in this adaptation. These adjustments in lipid metabolism can mainly be found in the proximal and middle part of the small intestine, where fat absorption commonly takes place. The lipid metabolism-related changes that were already found on the 30E% fat diet are even more pronounced on the 45E% fat diet. Additionally, other lipid metabolism-related genes were highly affected on the 45E% fat diet. Amongst those are genes that relate to reduced cholesterol absorption, which was previously suggested to be related to development of metabolic syndrome [18]. We also found other biological processes that are substantially affected on the 45E% fat diet, such as cell cycle and immune response-related processes. As the gene expression changes related to these processes are mainly restricted to the 45E% fat diet, these processes do not seem to directly contribute to the onset of the obese phenotype. However, they could be important for remodeling and adjusting immune functions in the small intestine. The intestinal overload of fat on the 45E% fat diet is also reflected in the increased numbers of differentially expressed genes in the middle and even the distal part of the gut. This may be taken as an indicator that fat digestion and absorption now extents to more distal parts that also adapts with changes in gene expression.

In summary, intestinal gene expression shows major changes by increasing the dietary fat content in mouse diets and numerous genes display a dose-dependent alteration in mRNA levels depending on dietary fat content. Genes induced by feeding 20E% and 30E% fat diets are predominantly related to lipid metabolism and especially to those regulated by Pparα. In the intestine of animals fed 45E% fat, changes in gene expression extend more into distal segments of the small intestine indicating compensatory adaptation to allow more fat to be digested and absorbed. At the highest fat intake, numerous genes with changed expression levels can be identified that relate to alterations in cell cycle and immune functions. From our study we may conclude that dietary fat itself has a pronounced effect on the intestinal transcriptome as indicated by dose-dependent changes in gene expression – mainly in the proximal and middle small intestine – and that especially alterations in lipid metabolism-related genes may contribute to obesity development.

## Supporting Information

Table S1 **Diet composition.** * Mineral Mix S10026 contains the following (g/kg mineral mix): magnesium oxide, 41.9; magnesium sulfate.7H2O, 257.6; sodium chloride, 259; chromium KSO4.12H2O, 1.925; cupric carbonate, 1.05; potassium iodate, 0.035; ferric citrate, 21; manganous carbonate, 12.25; sodium selenite, 0.035; zinc carbonate, 5.6; sodium fluoride, 0.20; ammonium molybdate.4H2O, 0.30; sucrose, 399.105. * Vitamin Mix V10001 contains the following (g/kg vitamin mix): retinyl palmitate, 0.80; cholecalciferol, 1.0; all-rac-a-tocopheryl acetate, 10; menadione sodiumbisulfite, 0.08; biotin (1.0%), 2.0; cyancocobalamin (0.1%), 1.0; folic acid, 0.20; nicotinic acid, 3.0; calcium pantothenate, 1.6; pyridoxine-HCl, 0.70; riboflavin, 0.60; thiamin-HCl, 0.60; and sucrose, 978.42.
(DOC)
