# Supplementary material for: Dose-Dependent Effects of Dietary Fat on Development of Obesity in Relation to Intestinal Differential Gene Expression in C57BL/6J Mice
Source: PLoS One. 2011 Apr 25;6(4):e19145. doi: 10.1371/journal.pone.0019145 (PMC3081848; doi:10.1371/journal.pone.0019145)
Supplement: Table S1 — Diet composition. * Mineral Mix S10026 contains the following (g/kg mineral mix): magnesium oxide, 41.9; magnesium sulfate.7H2O, 257.6; sodium chloride, 259; chromium KSO4.12H2O, 1.925; cupric carbonate, 1.05; potassium iodate, 0.035; ferric citrate, 21; manganous carbonate, 12.25; sodium selenite, 0.035; zinc carbonate, 5.6; sodium fluoride, 0.20; ammonium molybdate.4H2O, 0.30; sucrose, 399.105. * Vitamin Mix V10001 contains the following (g/kg vitamin mix): retinyl palmitate, 0.80; cholecalciferol, 1.0; all-rac-a-tocopheryl acetate, 10; menadione sodiumbisulfite, 0.08; biotin (1.0%), 2.0; cyancocobalamin (0.1%), 1.0; folic acid, 0.20; nicotinic acid, 3.0; calcium pantothenate, 1.6; pyridoxine-HCl, 0.70; riboflavin, 0.60; thiamin-HCl, 0.60; and sucrose, 978.42. (DOC) [file pone.0019145.s001.doc]

**Table S1. Diet composition**

|  | **10E% fat** | | **20E% fat** | | **30E% fat** | | **45E% fat** | |
| --- | --- | --- | --- | --- | --- | --- | --- | --- |
|  | **gm%** | ***kcal%*** | **gm%** | ***kcal%*** | **gm%** | ***kcal%*** | **gm%** | ***kcal%*** |
| Protein | 19 | *20* | 20 | *20* | 22 | *20* | 24 | *20* |
| Carbohydrate | 67 | *69* | 60 | *60* | 53 | *50* | 40 | *35* |
| Fat | 4 | *10* | 9 | *20* | 14 | *30* | 24 | *45* |
| Other | 10 | *1* | 11 | *1* | 11 | *1* | 12 | *1* |
| Total | 100 | *100* | 100 | *100* | 100 | *100* | 100 | *100* |
| kcal/gm | 3.8 |  | 4,1 |  | 4,3 |  | 4.7 |  |
|  |  |  |  |  |  |  |  |  |
| **Ingredient** | **gm** | ***kcal*** | **gm** | ***kcal*** | **gm** | ***kcal*** | **gm** | ***kcal*** |
| Casein, lactic | 200 | *800* | 200 | *800* | 200 | *800* | 200 | *800* |
| L-Cystine | 3 | *12* | 3 | *12* | 3 | *12* | 3 | *12* |
|  |  |  |  |  |  |  |  |  |
| **Corn Starch** | **427,2** | *1709* | **325,8** | *1303* | **224,3** | *897* | 72,8 | *291* |
| **Maltodextrin** | **100** | *400* | **100** | *400* | **100** | *400* | 100 | *400* |
| **Sucrose** | **172,8** | *691* | **172,8** | *691* | **172,8** | *691* | 172.8 | *691* |
|  |  |  |  |  |  |  |  |  |
| Cellulose, BW200 | 50 | *0* | 50 | *0* | 50 | *0* | 50 | *0* |
|  |  |  |  |  |  |  |  |  |
| Soybean Oil | 25 | *225* | 25 | *225* | 25 | *225* | 25 | *225* |
| **Palm oil** | **20** | *180* | **65,2** | *586* | **110,2** | *992* | **177.5** | *1598* |
|  |  |  |  |  |  |  |  |  |
| Mineral Mix S10026* | 10 | *0* | 10 | *0* | 10 | *0* | 10 | *0* |
| DiCalcium Phosphate | 13 | *0* | 13 | *0* | 13 | *0* | 13 | *0* |
| Calcium Carbonate | 5,5 | *0* | 5,5 | *0* | 5,5 | *0* | 5,5 | *0* |
| Potassium Citrate, 1 H2O | 16,5 | *0* | 16,5 | *0* | 16,5 | *0* | 16.5 | *0* |
|  |  |  |  |  |  |  |  |  |
| Vitamin Mix V10001* | 10 | *40* | 10 | *40* | 10 | *40* | 10 | *40* |
| Choline Bitartrate | 2 | *0* | 2 | *0* | 2 | *0* | 2 | *0* |
|  |  |  |  |  |  |  |  |  |
|  |  |  |  |  |  |  |  |  |
| **Total** | **1055** | ***4057*** | **999** | ***4057*** | **942** | ***4057*** | **858** | ***4057*** |
